# Analysis of posaconazole as oral antifungal prophylaxis in pediatric patients under 12 years of age following allogeneic stem cell transplantation

**DOI:** 10.1186/1471-2334-12-263

**Published:** 2012-10-19

**Authors:** Michaela Döring, Carsten Müller, Pascal-David Johann, Annika Erbacher, Astrid Kimmig, Carl-Philipp Schwarze, Peter Lang, Rupert Handgretinger, Ingo Müller

**Affiliations:** 1 Department of Pediatric Hematology and Oncology, University Children’s Hospital Tübingen, Hoppe-Seyler-Str.1, Tübingen, 72076, Germany; 2 Department of Therapeutic Drug Monitoring, Institute of Pharmacology, University of Cologne, Gleueler Str. 24, Cologne, 50931, Germany; 3 Department of Pediatric Oncology, Hematology and Immunology, University of Heidelberg, Im Neuenheimer Feld 430, Heidelberg, 69120, Germany; 4 Clinic of Pediatric Hematology and Oncology, University Medical Center Hamburg-Eppendorf, Martinistr. 52, Hamburg, 20246, Germany

**Keywords:** Posaconazole, Posaconazole trough levels, Pediatric patients, Antifungal prophylaxis, Stem cell transplantation

## Abstract

**Background:**

Pediatric patients undergoing hematopoietic stem cell transplantation (HSCT) are at high risk of acquiring fungal infections. Antifungal prophylaxis shortly after transplantation is therefore indicated, but data for pediatric patients under 12 years of age are scarce. To address this issue, we retrospectively assessed the safety, feasibility, and initial efficacy of prophylactic posaconazole in children.

**Methods:**

60 consecutive pediatric patients with a median age of 6.0 years who underwent allogeneic HSCT between August 2007 and July 2010 received antifungal prophylaxis with posaconazole in the outpatient setting. 28 pediatric patients received an oral suspension at 5 mg/kg body weight b.i.d., and 32 pediatric patients received the suspension at 4 mg/kg body weight t.i.d. The observation period lasted from start of treatment with posaconazole until its termination (maximum of 200 days post-transplant).

**Results:**

Pediatric patients who received posaconazole at 4 mg/kg body weight t.i.d. had a median trough level of 383 μg/L. Patients who received posaconazole at 5 mg/kg body weight b.i.d. had a median trough level of 134 μg/L. Both regimens were well tolerated without severe side effects. In addition, no proven or probable invasive mycosis was observed.

**Conclusion:**

Posaconazole was a well-tolerated, safe, and effective oral antifungal prophylaxis in pediatric patients who underwent high-dose chemotherapy and HSCT. Posaconazole at a dosage of 12 mg/kg body weight divided in three doses produced consistently higher morning trough levels than in patients who received posaconazole 5 mg/kg body weight b.i.d. Larger prospective trials are needed to obtain reliable guidelines for antifungal prophylaxis in children after HSCT.

## Background

Invasive fungal infection plays a major role in immunocompromised pediatric patients after high-dose chemotherapy and allogeneic hematopoietic stem cell transplantation (HSCT) [1-3]. Antifungal prophylaxis early after transplantation is therefore indicated, yet data for oral antifungal prophylaxis in pediatric patients who have undergone stem cell transplantation are scarce. In our clinic, the intravenous antifungal prophylaxis in pediatric HSCT recipients consisted of liposomal amphotericin B (1 mg/kg/day) during conditioning. On day +1 after HSCT we changed to caspofungin in a dosage of 1 x 50 mg/m^2^/day due to lower nephrotoxicity with comparable efficacy [4].

Posaconazole, a broad-spectrum triazole, has shown activity against *Candida spp., Aspergillus spp., Cryptococcus spp.,* and certain agents of mucormycosis and fusariosis in adults [5-8]. While most azole derivatives inhibit a broad range of cytochrome c-dependent enzymes and interfere with other drugs, including immunosuppressants, posaconazole is a potential inhibitor of CYP 3A4 and is not significantly metabolized by any CYP isozyme [9-11]. Posaconazole was more effective than fluconazole or itraconazole in prevention of invasive fungal infections in adult patients with myelodysplastic syndrome or acute myeloid leukemia [12]. It was superior in preventing invasive aspergillosis and reducing the rate of deaths related to fungal infections in adults with graft-versus-host disease [13]. A retrospective analysis presented evidence that long-term posaconazole prophylaxis in adults after HSCT was associated with few invasive mold infections [14].

The effectiveness and low incidence of side effects of posaconazole in adults and break-through infections with other azoles in our clinic was the rationale for changing to posaconazole for oral prophylaxis in pediatric patients. There was no drug licensed in Germany for antifungal prophylaxis in children after BMT at the time of this retrospective survey. To address specific questions in relation to pharmacokinetic evaluations, we conducted plasma level measurements. In this retrospective study, we analyzed the plasma concentrations of posaconazole during antifungal monoprophylaxis in two different posaconazole prophylaxis regimens. We herein present the safety, feasibility, and initial efficacy of antifungal prophylaxis with posaconazole after allogeneic HSCT in 60 pediatric patients under 12 years of age.

## Methods

### Study design

In this retrospective, single-center study we analyzed pediatric patients under 12 years of age who underwent allogeneic HSCT from August 2007 to July 2010 at the University Children’s Hospital Tübingen, Germany. This retrospective study was performed under the waiver for retrospective anonymized studies in accordance with the institutional ethics regulations. Drug levels were analyzed in excessive blood samples drawn for routine laboratory tests. Posaconazole was commenced upon discharge from the BMT unit following systemic antifungal prophylaxis with liposomal amphotericin B or caspofungin. We adapted the recommended dosage of 200 mg t.i.d. in adults (approximately equivalent to 10 - 12 mg/kg body weight in a adult weighing 50 - 60 kg) to the weight of pediatric patients [9]. Patients received posaconazole at either 5 mg/kg body weight b.i.d. or 4 mg/kg body weight t.i.d. Posaconazole was continued until day 100 following allogeneic HSCT and then until CD3^+^ T cells reached 200/μL and CD4^+^ T cells reached 100/μL in the peripheral blood as determined by flow cytometry. The observation period in this retrospective analysis was defined as the period from the start of posaconazole until treatment cessation and was no longer than day 200 after HSCT. The primary endpoint of this trial was the incidence of proven or probable invasive fungal infection during the observation period under posaconazole therapy. Criteria for diagnosis of invasive fungal infection were used as proposed by the Cooperative Group of the European Organization for Research and Treatment of Cancer and Mycoses Study Group of the National Institute of Allergy and Infectious Diseases (EORTC) [15]. Concerns about safety and tolerance provided secondary endpoints.

### Assessment of safety and tolerance

Treatment-related clinical and laboratory adverse events were registered, analyzed, and graded according to the current US National Cancer Institute’s Common Terminology Criteria for Adverse Events [16]. Blood analyses were recorded before, during, and at the end of antifungal prophylaxis. Blood samples were taken at least twice a week until day 100 and at least once a week thereafter until day 200. Baseline values were analyzed on the day before the start of posaconazole treatment.

### Assessment of efficacy

During the course of treatment with posaconazole, all pediatric patients were under continuous surveillance for proven or probable fungal infections. Monitoring took place via regular clinical assessment, routine laboratory analyses of factors including C-reactive protein and galactomannan antigen, and computed tomography, where indicated. At least once weekly until day 100 post-transplant, galactomannan antigen was measured by an *Aspergillus* enzyme-linked immunosorbent assay (Platelia™; Bio-Rad Laboratories, Munich, Germany). Breakthrough infection was defined as proven and probable invasive fungal infection according to EORTC criteria of 2008 [15]. Successful oral antifungal monoprophylaxis with posaconazole was defined as the absence of fungal infection during and at the end of oral prophylaxis as well as the absence of clinical and laboratory adverse events associated with posaconazole.

### Posaconazole plasma concentrations

By examining the excess material remaining after laboratory analyses, which had been routinely cryopreserved, we analyzed the trough plasma concentrations of posaconazole in six pediatric patients receiving posaconazole at 5 mg/kg body weight b.i.d. and eight pediatric patients receiving posaconazole at 4 mg/kg body weight t.i.d. by high-performance liquid chromatography [17].

### Statistical considerations

All data were analyzed by descriptive and inferential statistics. The pharmacokinetic analysis, which compared the measured posaconazole trough levels at the various dosages, was performed via the Mann-Whitney U test. The Wilcoxon matched-pairs signed-ranks test was used for statistical comparison of continuous blood parameters, transaminases, and cholestasis parameters. To determine whether our sample group exhibited statistically significantly different values from the age-adjusted normal range, the one-sample t-test, taking into account 95% confident intervals, was used. Analysis of the influence of posaconazole on the CsA serum concentration was first performed with the Friedman two-way analysis of variance by ranks, then secondarily with the Wilcoxon matched-pairs signed-ranks test, using four comparison points: days 2 to 3, days 4 to 6, days 8 to 12, and days 16 to 20 after the start of posaconazole. P values of ≤ 0.05 (*), ≤ 0.01 (**), and ≤ 0.001 (***) were defined as statistically significant. Graphs were created with the graphics program GraphPad Prism 4 for Windows, version 4.03 (GraphPad Software, San Diego, CA, USA), and statistical analysis was carried out with the statistical program XLSTAT 2010 (Addinsoft, Paris, France).

## Results

### Patient characteristics

We assessed 60 consecutive pediatric patients (38 males, 22 females) under 12 years of age with hemato-oncological malignancies and inborn errors of metabolism who received primary oral antimycotic monoprophylaxis with posaconazole after allogeneic HSCT. Posaconazole was given in the outpatient setting and initiated two to four days before discharge. The most common primary diagnoses were acute lymphoblastic leukemia in 17 (28.3%) and aplastic anemia in 9 (15%) of 60 pediatric patients. The median age was 6.0 years (range, 0.7 - 11.5 years). 30 of the 60 pediatric patients received a myeloablative conditioning regimen (MAC), from which 12 of 30 received TBI, 2 of 30 were treated with busulfan, and 16 of 30 with treosulfan. 20 of the 60 pediatric patients received a reduced-intensity conditioning regimen (RIC) with melphalan. 5 patients received a conditioning regimen with fludarabin and thiotepa, 4 patients with fludarabin and cyclophosphamide, and 1 patient with thiotepa and cyclophosphamide. Patients receiving a graft from a MUD, MMUD or MFD received short course MTX and CsA. Patients undergoing haploidentical transplantation received a T-cell depleted graft and mycophenolate mofetil only. All Patients with GvHD listed in Table 1 were treated.

**Table 1:** Patient characteristics

**Characteristic**	**No. (%) (n = 60)**
Gender	
Male	38 (63.3)
Female	22 (36.7)
Age group	
< 6 years	26 (43.3)
6-11 years	34 (56.7)
Donor	
MUD	20 (33.3)
MMFD	27 (45.0)
MFD	13 (21.7)
Primary diagnosis	
ALL	6 (10.0)
ALL relapse	11 (18.3)
AML	2 (3.3)
AML relapse	2 (3.3)
Biphenotypic leukemia	1 (1.7)
CML	2 (3.3)
JMML	3 (5.0)
MDS	5 (8.3)
Solid tumors	7 (11.7)
Aplastic anemia	9 (15.0)
Neurometabolic diseases	4 (6.7)
Immunologic diseases	6 (10.0)
Chediak-Higashi-syndrome	1 (1.7)
Kostmann disease	1 (1.7)
Graft-versus-Host-Disease	
Grade I	23 (38.3)
Grade II	3 (5.0)
Grade III	4 (6.7)
Grade IV	2 (3.3)
Chronic limited	2 (3.3)
Chronic extensive	1 (1.7)

Abbreviation: ALL, acute lymphoblastic leukemia; AML, acute myelogenous leukemia; CML*,* chronic myeloid leukemia; JMML, juvenile myelomonocytic leukemia; MDS*,* myelodysplastic syndromes*,* MFD, matched family donor; MMFD, mismatched family donor; MUD, matched unrelated donor.

Twenty-eight pediatric patients received an oral suspension of posaconazole at 5 mg/kg body weight b.i.d., and the remaining 32 patients received the suspension at 4 mg/kg body weight t.i.d. Not any other changes to the allogeneic HSCT protocol or prophylaxis were made simultaneously with the change in posaconazole dosing. The median observation period for the treatment with posaconazole lasted until day 162 after allogeneic HSCT (with a range from day 24 to day 212 after HSCT). The median treatment period with posaconazole was 127 days (range 12 - 188 days). Eight of the 60 pediatric patients were monitored for a period less than one hundred days after HSCT (with a range from 24 to 86 days after HSCT). The intravenous antifungal prophylaxis before the start of posaconazole was liposomal amphotericin B in 23 patients and caspofungin in the remaining 37. Children received cotrimoxazole and aciclovir simultaneously with posaconazole.

The median duration of neutropenia pre-engraftment was 12 days (range 10 - 24 days), mean 12.58 ± 1.84. Because of post-transplant complications in two patients with intestinal graft-versus-host disease and two patients with severe bacterial infection, oral posaconazole was replaced by intravenous caspofungin. These patients were included in the analysis until change of the antifungal prophylaxis.

### Efficacy analysis

All 60 pediatric patients were included in the final efficacy analysis. There were no incidences of proven or probable invasive fungal infection during treatment with posaconazole as antifungal prophylaxis. Five patients (8.3%) died of causes other than invasive fungal infection, including relapse of the underlying disease in four cases, and a single case of disseminated intravascular coagulation (DIC). Two of them died during the observation period with posaconazole. These patients were included in the analysis until antifungal prophylaxis with posaconazole was discontinued.

### Safety and tolerability analysis

Side effects potentially related to posaconazole were recorded in five (8.3%) of the 60 patients; these included one patient with pruritus (1.7%), three with nausea (5%), and one with vomiting (1.7%) (Table 2). All of these secondary effects were of severity grade I or II [16]. In the cases of vomiting (n = 1) and nausea (n = 3), we ceased the antifungal prophylaxis with posaconazole and continued with voriconazole, instead. These patients were included in the analysis up until the last day of their treatment with posaconazole.

**Table 2:** Clinical and laboratory adverse events of antifungal prophylaxis

**Variable**	**No. (%) (n = 60)**
Drug related adverse events	
Clinical (total)	5 (8.3)
Fever	0 (0)
Pruritus	1 (1.7)
Nausea	3 (5.0)
Vomiting	1 (1.7)
Increase in alanine aminotransferase	
≥ 2.0 x normal value 39 U/L	10 (16.7)
≥ 3.0 x normal value 39 U/L	9 (15)
Increase in aspartate aminotransferase	
≥ 2.0 x normal value 59 U/L	1 (1.7)
≥ 3.0 x normal value 59 U/L	0 (0)
Increase in total bilirubin	
≥ 2.0 x normal value 1.1 mg/dl	0 (0)
≥ 3.0 x normal value 1.1 mg/dl	0 (0)
Increase in direct bilirubin	
≥ 2.0 x normal value 0.3 mg/dl	2 (3.4)
≥ 3.0 x normal value 0.3 mg/dl	0 (0)
Analysis of cyclosporin A level	(n = 28)
≥ 1.5 x baseline day 2-3	8 (28.6)
≥ 2.0 x baseline day 2-3	2 (7.1)
≥ 1.5 x baseline day 4-6	9 (32.1)
≥ 2.0 x baseline day 4-6	1 (3.6)
≥ 1.5 x baseline day 8-12	4 (14.3)
≥ 2.0 x baseline day 8-12	1 (3.6)
≥1.5 x baseline day 16-20	1 (3.6)
≥ 2.0 x baseline day 16-20	0 (0.0)

**Figure 1:**
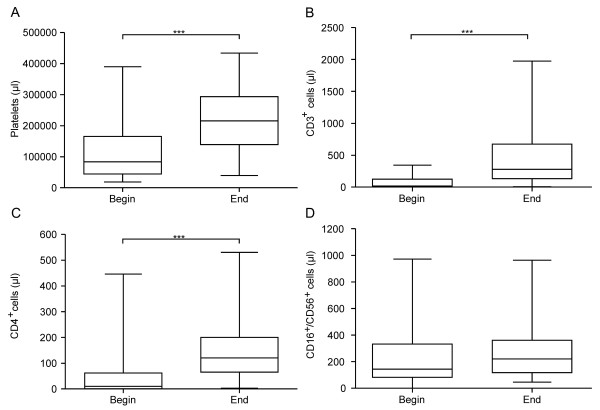
**Blood analyses.** Box plot of peripheral blood cell count values the day before posaconazole therapy began (Begin) and at the end of oral antimycotic monoprophylaxis with posaconazole (End). **A**: Platelet counts. **B**: CD3^+^ cell counts. **C**: CD4^+^ cell counts. **D**: CD16^+^/CD56^+^ cell counts. A timely reconstitution of platelets (***P < 0.001), CD3^+^ cell counts, and CD4^+^ cell counts took place during posaconazole treatment.

**Figure 2:**
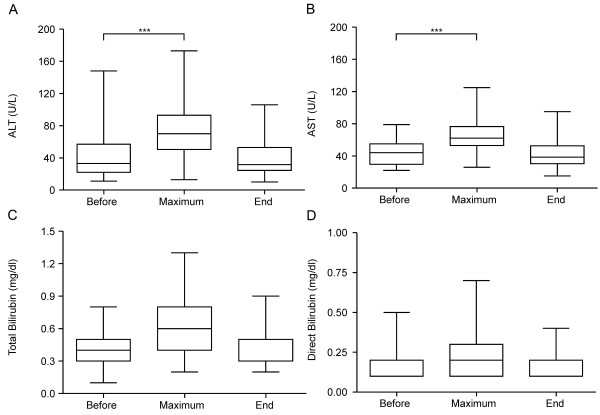
**Hepatotoxicity.** Box plot of liver parameters the day before posaconazole therapy began (Before), their maximums during therapy (Maximum), and values at the end of oral antimycotic monoprophylaxis with posaconazole (End). **A**: Plasma alanine aminotransferase (ALT) (normal value, ≤ 39 U/L). **B**: Plasma aspartate aminotransferase (AST) (normal value, ≤ 59 U/L for pediatric patients < 12 years of age). **C**: Total plasma bilirubin (normal value, ≤ 1.1 mg/dL). **D**: Direct plasma bilirubin (normal value, ≤ 0.3 mg/dL). Statistical analysis by the Wilcoxon matched-pairs signed-ranks test showed a significant increase (***P < 0.001) in ALT and AST beyond their upper normal limits during treatment post-transplant respective to baseline measurements taken the day before the start of posaconazole prophylaxis (Before).

Reconstitution of hematopoiesis, including platelets and lymphocyte subpopulations, proceeded in a timely manner (Figure 1). Hepatic function testing showed significant grade I and II alanine aminotransferase (ALT) elevations (P < 0.001) and grade I aspartate aminotransferase (AST) elevations (P < 0.001) during posaconazole treatment respective to baseline (Figure 2). In 84% of patients, these increases emerged by day 14 after the start of treatment. Interestingly, these parameters normalized in 86% of patients by day 27 after the start of posaconazole. The significant increases (P < 0.001) in ALT and AST were observed in both treatment groups (both b.i.d. and t.i.d. posaconazole therapy). Cholestasis parameters, such as direct bilirubin, increased during posaconazole treatment in two patients with grade II elevations (one patient in each treatment group).

### Cyclosporin A levels

Analysis of cyclosporin A (CsA) whole blood concentrations was performed during the observation period in 28 pediatric patients treated with CsA + posaconazole. A total of 16 (57.1%) pediatric patients exhibited a statistically significant yet moderate increase in CsA blood concentration (by 28%) on days 2 to 6 (P < 0.01) after the start of posaconazole (Table 2), and a reduction in the CsA dose, which ranged from 10% to 32%, was performed in 12 (75.0%) of these 16 patients at that point. Adjustments to the dosage of CsA after treatment day 8 took place in only one of the twelve (8.3%) patients. An average CsA dose reduction of 22% (range, 10 - 32%) was sufficient to compensate for these drug interactions.

### Posaconazole levels

**Figure 3:**
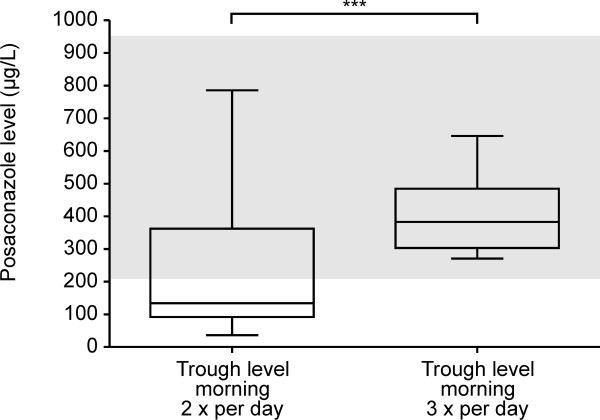
**Posaconazole trough levels.** Analysis of posaconazole morning trough levels at 5 mg/kg body weight daily (b.i.d.) (n = 48) and morning trough levels at 4 mg/kg body weight daily (t.i.d.) (n = 41). These morning trough levels were significantly different (P < 0.001) according to the Mann-Whitney U test. The gray area indicates the reported range of posaconazole trough levels in adults.

A total of 48 trough plasma concentrations of posaconazole were analyzed in 6 pediatric patients who had received an oral suspension of posaconazole at 5 mg/kg body weight b.i.d., and a total of 41 trough plasma concentrations of posaconazole were analyzed in 8 pediatric patients who had received an oral suspension of posaconazole at 4 mg/kg body weight t.i.d. Demographic characteristics of these 14 pediatric patients are presented in Table 3. Pediatric patients who received posaconazole t.i.d. had consistently higher morning trough levels (median, 383 μg/L; range, 61 - 1243 μg/L; mean, 434.81 ± 241.01 μg/L) in the observation period from day 3 to 164 after starting posaconazole compared with patients who received posaconazole b.i.d. (median, 134 μg/L; range, 56 - 786 μg/dl; mean, 221.4 ± 188.8 μg/L) in the observation period from day 3 to 184 after the start of treatment. These morning trough levels were significantly different (P < 0.001) according to the Mann-Whitney U test (Figure 3). There were no significant differences in the trough plasma concentrations of posaconazole between patients < 6 years of age and patients 6 to 11 years of age, who had received posaconazole at 5 mg/kg body weight b.i.d. (P = 0.36) and patients who had received posaconazole at 4 mg/kg body weight t.i.d. (P = 0.77). In terms of food intake, the mean posaconazole trough level (n = 28) was 33.5% to 63.5% higher in children who received posaconazole during a fatty meal (e.g., a meal including a spoon of coffee cream and posaconazole) compared with the mean trough level in children who received posaconazole with a meal without coffee cream or, in some cases, without any food at all. The trough levels (n = 13) in the two patients who received posaconazole b.i.d. with a fatty meal (median, 330 μg/L; mean, 319.6 ± 187.13 μg/L) were significant higher (P = 0.025) than the trough levels (n = 35) in four patients who received posaconazole b.i.d. with a normal meal without coffee cream or without any food at all (median, 133 μg/L; mean, 199.63 ± 181.08 μg/L). In three patients who received posaconazole t.i.d. with a fatty meal, the posaconazole trough levels (n = 15) (median, 467 μg/L; mean, 450 ± 129.71 μg/L) were higher than the trough levels (n = 26) in five patients who received posaconazole with a normal meal (median, 361 μg/L; mean, 337 ± 127.25 μg/L), but the difference was not significant (P = 0.09). The first measured posaconazole trough levels early after the start of posaconazole treatment between days 3 and 6 were lower in both treatment regimen groups. The mean posaconazole trough level increased significantly (P = 0.049) between days 3 and 6 (median, 91 μg/L; mean, 112.71 ± 91.42 μg/L) and after day 7 (median, 136 μg/L; mean, 230.6 ± 197.37 μg/L) in patients who received posaconazole b.i.d. Patients who received posaconazole t.i.d. showed lower trough levels between days 3 and 6 (mean, 302.25 ± 183.67 μg/L; median, 330 μg/L; range, 61 - 488 μg/L) than those after day 7 (mean, 476.21 ± 246.87 μg/L; median, 404 μg/L; range, 291 - 1243 μg/L), but the difference was not significant (P = 0.27). Seventy-nine (88.7%) of all (n = 89) posaconazole trough levels in this retrospective analysis were measured during the use of H2 receptor antagonist ranitidine or the proton pump inhibitor omeprazole. Three of 24 pediatric patients in whom posaconazole trough levels were measured experienced diarrhea. These cases consisted of two of the children who had received posaconazole three times daily and one child who had received posaconazole twice daily. In none of these children did the levels lay lower than the trough levels of the children in the same group without diarrhea.

**Table 3:** Characteristics of patients with posaconazole level monitoring

**Characteristic**	**Posaconazole administration**	
	**2 x per day (n = 6)**	**3 x per day (n = 8**)
	**No. of patients (%)**	
Gender		
Male	2 (33.3)	5 (62.5)
Female	4 (66.6)	3 (37.5)
Age group		
< 6 years	3 (5.0)	6 (75.0)
6-11 years	3 (5.0)	2 (25.0)
Donor		
MUD	2 (33.3)	3 (37.5)
MMFD	2 (33.3)	5 (62.2)
MFD	2 (33.3)	0 (0)
Primary diagnosis		
ALL	1 (16.7)	2 (25.0)
Biphenotypic leukemia	0 (0)	1 (12.5)
Solid tumors	1 (16.7)	0 (0)
Aplastic anemia	2 (33.3)	1 (12.5)
Neurometabolic diseases	1 (16.7)	1 (12.5)
Immunodeficiency	1 (16.7)	3 (37.5)
Graft-versus-Host disease		
Grade I	2 (33.3)	2 (25.0)
Grade II	0 (0)	2 (25.0)
Grade III	0 (0)	0 (0)
Grade IV	0 (0)	0 (0)
Chronic limited	0 (0)	0 (0)
Chronic extensive	0 (0)	0 (0)

Abbreviation: ALL, indicates acute lymphoblastic leukemia; MFD, matched family donor; MMFD, mismatched family donor; MUD, matched unrelated donor.

## Discussion

No drugs were licensed in Germany for use as antifungal prophylaxis in children after BMT at the time when this retrospective survey took place. Furthermore, we had observed breakthrough infections with the treatment of azoles other than posaconazole in the past. In a historical group of 50 pediatric patients whom we had treated in our center with an antifungal prophylaxis with itraconazole after allogeneic HSCT, three possible fungal infections were observed. This data is in concordance with data obtained at other centers [18]. These reasons prompted our choice of posaconazole, due to its promising results in adult patients. To address specific questions with regard to pharmacokinetic evaluations, we conducted plasma level measurements. According to the current guidelines for antifungal management in adult patients, posaconazole is recommended as primary antifungal prophylaxis following allogeneic HSCT [19]. Based on the data on posaconazole as oral antifungal prophylaxis in adults we decided to use posaconazole in pediatric patients as well [12,13].

We retrospectively assessed data on the plasma levels, efficacy, safety, and tolerability of posaconazole in 60 pediatric patients under 12 years of age after HSCT. Varying dosages of posaconazole as antifungal prophylaxis and therapy for invasive fungal infections have been reported in adults [5,12], while dosages for pediatric patients have not yet been defined [20,21]. We adapted the recommended prophylactic dosage of 600 mg/day in adults to the weight of pediatric patients [9]. Initially, the frequency of administration of posaconazole was 5 mg/kg b.i.d. However, data became available showing that trough levels of pediatric patients were lower and less stable compared with those reported in adults [12,13,22,23]. Therefore, the frequency of administration and daily dosage were increased to 4 mg/kg t.i.d.

Posaconazole trough levels under the latter regimen were consistently higher than trough levels in patients who received posaconazole b.i.d. and only slightly lower than posaconazole trough levels in adults undergoing posaconazole prophylaxis [12,13]. In another report posaconazole levels > 0.5 mg/L correlated with a better outcome [24]. Although we mostly found lower values in our patient cohort, no invasive fungal infections were observed. This illustrates that a prospective randomized controlled study in pediatric patients is of pressing importance to verify this data. There is only one published pediatric study on dose determination involving a small group of 12 pediatric and juvenile patients (median age, 15 years) with invasive fungal infections; the therapeutic dose of posaconazole in 11 of 12 patients was 800 mg/day in divided doses with a median posaconazole through level of 579 ng/mL (range, 85.3 - 2891 ng/mL; mean, 776 ng/mL) [25]. In our prophylaxis study, we observed lower posaconazole trough levels (median, 404 ng/mL; range, 291 - 1243 ng/mL; mean, 476 ± 246.84 ng/mL) from day 7 after the start of treatment at 4 mg/kg t.i.d. A randomized crossover study with healthy subjects showed the highest posaconazole levels during and after a high-fat meal and with avoidance of proton pump inhibitors [26]. Most of the pediatric patients enrolled in our retrospective analysis received a proton pump inhibitor (76.67%, n = 46) or a H2 receptor antagonist (10%, n = 6) at least once a day during analysis of posaconazole trough levels; it is probable that the trough levels would be higher with avoidance of proton pump inhibitors.

Only a few studies have been published on the usage of posaconazole in pediatric patients with a small number of cases. A multi-center retrospective survey reported data on 15 pediatric patients (median age, 10 years) with invasive fungal infections, most of them with hemato-oncological malignancies, who received posaconazole salvage therapy at a median dosage of 21 mg/kg body weight (range, 4.8 - 33.3 mg/kg). Posaconazole was effective as salvage therapy for proven and probable invasive fungal infections [20]. Another retrospective survey on the off-label use of posaconazole as secondary prophylaxis and rescue therapy included 15 pediatric patients (median age, 10 years). Twelve of these patients experienced an invasive fungal infection, i.e. proven aspergillosis in one patient, probable aspergillosis in ten patients and possible mycosis in one patient. Three patients received posaconazole as primary (n = 2), and secondary (n = 1) prophylaxis in high-risk situations, i. e. treatment with steroids, acute GvHD, and haploidentical HSCT. 9 of the 15 patients were post-HSCT. Patients received varying dosages, including 200 mg/day (t.i.d.) in 12 cases, 400 mg/day (b.i.d.) in two cases, and 100 mg/day (t.i.d.) in a single patient [21]. Posaconazole was well tolerated and showed significant clinico-radiological improvements in 9 of these 12 patients. In our pediatric patients, we observed a significant but moderate and reversible increase in the liver parameters ALT and AST. This finding corresponds with observations made in a retrospective study of salvage therapy in pediatric patients [20]. The survey also reported side effects similar to those in our analysis, such as nausea and vomiting. Drug interactions involving posaconazole and calcineurin inhibitors can occur due to inhibition of the CYP3A4 isoenzyme [11]. We observed a significant increase in the CsA plasma concentration in 16 (57.1%) of the 28 pediatric patients in our cohort. An average CsA dose reduction of 22% (range, 10 - 32%) was sufficient to adjust for this reaction. Similar elevated CsA trough levels were also observed in up to 41% of patients in another retrospective review of adult and pediatric HSCT recipients after voriconazole administration [27]. The limitations of the measured posaconazole levels in our analysis are that they took place retrospectively from excess material remaining after laboratory analyses which had been routinely cryopreserved, and that only on arbitrary time points after day seven (the point commonly known to be the time when a steady state of posaconazole levels plasma is reached) were possible. It is of utmost importance to establish a pharmacokinetic analysis within the framework of a prospective, randomized study in pediatric patients.

## Conclusions

We retrospectively studied posaconazole as oral antifungal prophylaxis in the largest published cohort of post-HSCT pediatric patients under 12 years of age to date. We conclude that posaconazole at a dosage of 4 mg/kg body weight t.i.d. with a fatty meal is an effective, safe, and well-tolerated antifungal prophylactic regimen in these pediatric patients. We observed a low incidence of side effects and continually stable posaconazole serum concentrations. Because of our favorable experience with posaconazole as antifungal prophylaxis in pediatric patients after allogeneic HSCT, we are currently using this regimen at a dosage of 4 mg/kg body weight t.i.d. Prospective clinical trials in pediatric patients are needed to compare posaconazole with other antifungals and identify drug interactions in this cohort.

## Abbreviations

b.i.d: Bis in die, twice a day; t.i.d: Ter in die, three times a day; CsA: Cyclosporin A.

## Competing interests

All authors declared that they have no competing interest.

## Authors’ contributions

MD has made substantial contributions to conception and design, acquisition of data, analysis and interpretation of data and wrote the paper. CM has made substantial contributions to conception and design, analysis and interpretation of data. PDJ has been involved in collection of data. AE participated in the analysis and interpretation of data. AK and CPS have been involved in acquisition of funding and collection of data. PL and RH have been involved in revising the manuscript critically for important intellectual content. IM has made substantial contributions to conception, design, analysis and interpretation of data and has given final approval of the version to be published. All authors read and approved the final manuscript.

## Pre-publication history

The pre-publication history for this paper can be accessed here:

http://www.biomedcentral.com/1471-2334/12/263/prepub
